# Factors associated with treatment success after combined transforaminal epidural steroid injection and dorsal root ganglion pulsed radiofrequency treatment in patients with chronic lumbar radicular pain

**DOI:** 10.55730/1300-0144.5429

**Published:** 2022-06-26

**Authors:** Gülçin GAZİOĞLU TÜRKYILMAZ

**Affiliations:** Department of Pain Medicine, Bursa City Hospital, Bursa, Turkey

**Keywords:** Transforaminal epidural steroid injection, dorsal root ganglion, pulsed radiofrequency treatment, lumbar radicular pain, numeric rating scale

## Abstract

**Background/aim:**

The aim of the study is to identify predictors of treatment success with combined transforaminal epidural steroid injection (TFESI) and dorsal root ganglion pulsed radiofrequency (DRG-PRF) in patients with lumbar radicular pain (LRP) associated with lumbar disc herniation.

**Materials and methods:**

The study included 48 patients with herniation-related LRP who underwent TFESI and DRG-PRF treatment between November 1, 2020 and April 30, 2021. Patient age, sex, symptom duration, history of lumbar surgery, and numeric rating scale (NRS) pain scores before and at 10 days, 1 month, and 3 months after treatment were evaluated retrospectively. Treatment success was defined as ≥50% improvement or a 4-point decrease in NRS score at 3 months.

**Results:**

Twenty-nine female and 19 male patients with a mean age of 51.54 ± 13.31 years were analyzed. The median symptom duration was 6 (interquartile range: 8.50) months. Symptom duration did not affect treatment success (p = 0.105). History of spinal surgery was more common among patients with failed treatment but was not statistically associated with treatment success. A 1-unit increase in pre-treatment NRS score was associated with 72% lower odds of treatment success (p = 0.022), while a 1-unit increase in NRS score on post-treatment day 10 compared to the pre-treatment value was associated with 95% lower odds of treatment success (p = 0.008).

**Conclusion:**

Symptom duration and history of spinal surgery were not predictive of treatment success with combined TFESI and DRG-PRF for herniation-related LRP. However, the 3-month prognosis was significantly better for patients with a marked reduction in NRS score at 10 days.

## 1. Introduction

Lumbar radicular pain (LRP) can occur as a result of mechanical compression of the dorsal root or ganglion by herniated disc material or due to inflammation caused by chemokines and enzymes in the disc [1]. Studies have shown that transforaminal epidural steroid injections (TFESI) and dorsal root ganglion pulsed radiofrequency (DRG-PRF) interventions are effective when conservative treatment methods (e.g., oral analgesic drugs, exercise, and physiotherapy) are insufficient [2,3].

For LRP secondary to lumbar disc herniation (LDH), there are reports indicating the short-term efficacy of TFESI and DRG-PRF interventions when performed separately [3,4]. In addition, there are also studies demonstrating the efficacy of applying TFESI and DRG-PRF simultaneously [2,5]. Despite evidence that these methods are effective, successful treatment results are not obtained in all patients. Several studies have investigated the potential relationship between clinical parameters and the results of TFESI and DRG-PRF when used separately. Different results regarding age and symptom duration were reported in the studies examining the effect of clinical features on the success of TFESI [6,7], while age and history of failed lumbar surgery were reported to be factors affecting the success of DRG-PRF therapy [8,9].

A literature search yielded no study investigating clinical factors affecting treatment success with combined TFESI and DRG-PRF therapy for LRP. The aim of this study was to investigate the clinical factors associated with treatment success after combined TFESI and DRG-PRF therapy in patients with LRP due to LDH.

## 2. Materials and methods

### 2.1. Study design

This was a retrospective study.

### 2.2. Setting

Ethics committee approval for this study was obtained from the Clinical Research Ethics Committee of Bursa City Hospital (date: 02.06.2021, decision number: 2021-10/12). The study included patients who had LDH and associated chronic radicular pain that did not respond to conservative treatment consisting of medication and/or physical therapy and underwent combined TFESI and DRG-PRF between November 1, 2020 and April 30, 2021 after examination and imaging by the algology department. Patients whose symptom duration, surgical history, or numeric rating scale (NRS) pain scores were not recorded were excluded from the study.

### 2.3. Lumbar radicular pain clinical assessment and follow-up protocol

The protocol for combined TFESI and DRG-PRF in our clinic specifies that this intervention is performed at the vertebral level(s) causing symptoms in patients meeting the following criteria:

- Insufficient analgesia despite the use of at least one conservative treatment method,
- LRP persisting for at least 3 months,
- NRS value of 6 or higher at initial evaluation,
- Lumbar magnetic resonance imaging (MRI) demonstrating disc herniation without sequestration at a level consistent with physical examination findings.

At the patient’s first examination, a detailed history including symptom duration and history of previous lumbar surgery is recorded. Patients are asked to rate their pain intensity using the NRS before the procedure and at 10 days, 1 month, and 3 months after the procedure. Patients are also asked not to change their previously prescribed medications for at least 1 month after the procedure.

### 2.4. Procedures

**Figure 1:**
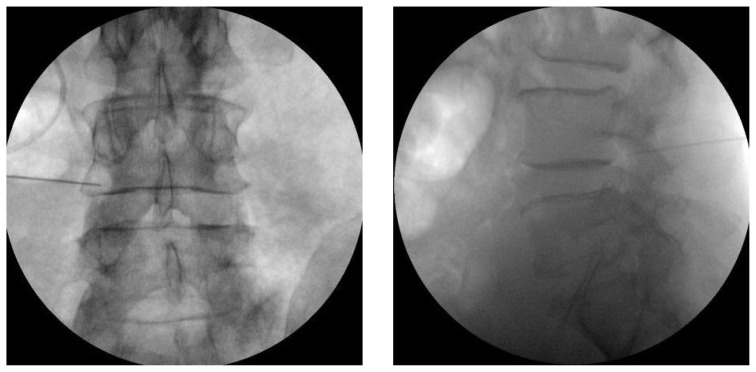
Position of the needle in AP and lateral view during DRG PRF.

**Figure 2:**
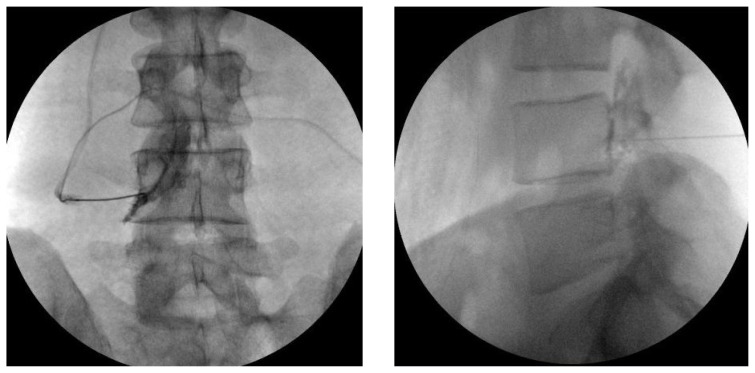
Position of the needle in AP and lateral view during TFESI.

All patients were evaluated by a single algologist and all TFESI and DRG-PRF procedures were performed by the same physician (G.T.). The procedures are performed under sterile conditions and mild sedation with standard monitoring. The patient is placed in prone position with a pillow under the lower abdomen to provide easy access to the intervertebral foramen. Under anterior-posterior (AP) fluoroscopy, the C-arm is moved cranially or caudally to the lower endplate of the vertebra at the target level. The C-arm is adjusted to a 25–30 degree ipsilateral oblique angle and skin infiltration with 1 cc 2% lidocaine is administered. A 10-cm 22-gauge RF needle with 10-mm active tip (TOP, Japan) is advanced as far medial to the intervertebral foramen and near the DRG as possible using a tunnel vision technique. The needle is positioned so as not to pass the middle of the intervertebral foramen in the lateral view and the middle of the pedicle column in AP and lateral view (Figure 1). After properly positioning the RF needle, a radiofrequency device (TOP-TLG 10 STP) is used to ensure the impedance readings in the PRF generator are <400 ohms. With sensory stimulation applied at 50 Hz, paresthesia is expected in the area corresponding to the distribution of the patient’s LRP at a voltage of <0.6 V. After applying 2 Hz motor stimulation, a confirmatory motor response is sought at 1.5 times the sensory threshold [2, 5]. After obtaining the appropriate responses, PRF is performed at 45 V and 42 °C for 120 s. After the DRG-PRF procedure, the needle is withdrawn by 2–3 mm to reposition the tip in the safe triangle. Contrast agent (Omnipaque 300, GE Healthcare, Ireland) is injected to confirm epidural spread, then a solution of 4 mg dexamethasone in 4 cc saline is administered in each target level (Figure 2).

### 2.5 Assessment

After 3 months, ≥ 50% improvement or a 4-point decrease in NRS score was considered a successful outcome. The need for alternative treatment and additional analgesic therapy in the first month of follow-up was regarded as nonresponse (treatment failure) [2].

## 3. Statistical analysis

In the present study, posthoc power analysis was performed based on NRS scores at 3 months after treatment. The mean NRS score was 5.63 ± 1.45 for patients whose treatment was unsuccessful (n = 16) and 2.09 ± 0.82 in those whose treatment was successful (n = 32). The effect size was calculated as d = 3.01. With this effect size, the power of the study at an alpha error level of 0.05 was determined as >95%. Power analysis calculations were made using the G*Power program (Faul, F., Erdfelder, E., Lang, A.-G., & Buchner, A. (2007). G*Power 3: A flexible statistical power analysis program for the social, behavioral, and biomedical sciences. Behavior Research Methods, 39, 175–191.)

Shapiro–Wilk test was used to evaluate whether age, symptom duration, and NRS score showed normal distribution. Based on the results, age was presented as mean and standard deviation, while symptom duration and NRS scores were expressed as median and interquartile range. Independent samples t-test was used to compare age, and Mann–Whitney U test was used to compare symptom duration and NRS values between patients with treatment success and failure. Comparisons of categorical variables between patient subgroups were performed with Fisher–Freeman–Halton and chi-square tests. Statistical analyses were performed using IBM SPSS Statistics version 21.0 for Windows (IBM Corporation, Armonk, NY, USA). Type I error level was accepted as 5% for all analyses.

Logistic regression analysis was used to identify factors associated with treatment success. The variables in Table 1 were first evaluated using univariate logistic regression analysis, and those with p < 0.25 were included in the multivariate logistic regression model.

## 4. Results

A total of 55 patients underwent the combined TFESI and DRG-PRF procedure due to LRP in the 5-month study period. Of these, 7 patients were excluded from the study because NRS values were not available for the 3-month follow-up period. The study group included 29 women and 19 men with a mean age of 51.54 ± 13.31 years. The mean duration of symptoms before injection was 6 months. Injections were most frequently applied at the level of L5–S1 (47.9%) and on the left (56.3%) side. The proportion of patients with no history of previous spinal surgery was 64.6%.

Based on NRS scores, a successful result was obtained in 66.6% of the patients (n = 32). The demographic characteristics and treatment details of patients with successful and unsuccessful treatment outcomes are shown in Table 1.

**Figure 3:**
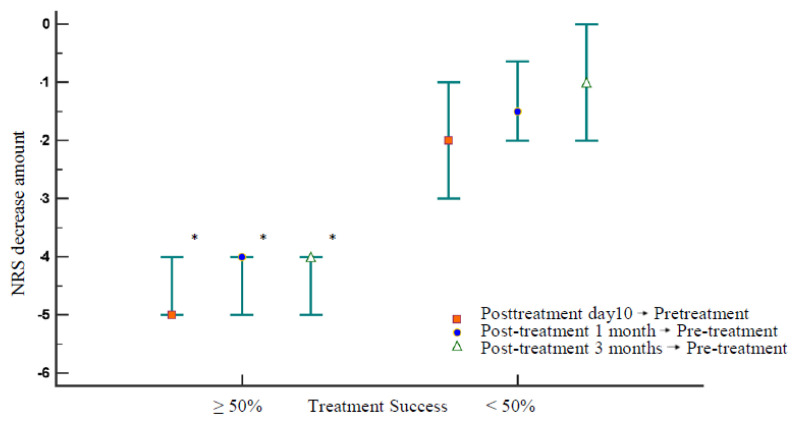
Relationship between treatment success and reduction in pain scores at 10 days, 1 month, and 3 months after the treatment (*p < 0.001).

There was no significant difference in age, sex, symptom duration, injection level and side, and pretreatment NRS scores between patients with successful and failed TFESI and DRG-PRF therapy (p > 0.05). However, there was a significant difference between patients with treatment success and failure in terms of spinal surgery history (p < 0.001). Subgroup analyses revealed that the treatment success rate was higher in patients with no history of spinal surgery compared to the patients with one or more spinal surgeries (p < 0.05). A greater decrease in NRS at 10 days, 1 month, and 3 months after treatment compared to the pre-treatment NRS value was observed in patients whose treatment was successful (p < 0.001 for all) (Figure 3).

Multivariate logistic regression analysis was performed including symptom duration, history of spinal surgery, pretreatment NRS, and change in NRS on posttreatment day 10 compared to pretreatment values. The results of the analysis are presented in Table 2.

The logistic regression model was statistically significant (p < 0.001) and fit the data (p = 0.987). Treatment success was not associated with symptom duration (p = 0.105), history of spinal surgery, or number of previous spinal surgeries. However, an increase of 1 unit in pretreatment NRS score was associated with 72% lower odds of treatment success (p = 0.022). In addition, a 1-unit increase in NRS score on posttreatment day 10 compared to the pretreatment value was associated with 95% lower odds of treatment success (p = 0.008).

A comparison of patients with and without a history of spinal surgery in terms of age, symptom duration, and NRS values is shown in Table 3.

There was no significant difference between patients with and without a history of spinal surgery in terms of mean age, median symptom duration, or pretreatment NRS values (p > 0.05). The reductions in NRS values at posttreatment 10 days, 1 month, and 3 months compared to pretreatment values were significantly greater at all time points in patients with no history of spinal surgery (p < 0.001 for all).

## 5. Discussion

In this study, combined TFESI and DRG-PRF therapy was successful in 66.6% of patients with LRP related to LDH. Patients with successful treatment outcomes at 3 months showed a significantly greater reduction in NRS pain scores 10 days after treatment. Although a history of spinal surgery was less common among patients with treatment success compared to patients with failed treatment, the results of multivariate analysis showed that history of spinal surgery did not significantly affect treatment success.

Administering TFESI to the ventral epidural region, which is the site of pathological changes, is a targeted and appropriate treatment option for herniation-related LRP. A metaanalysis examining the efficacy of TFESI in this patient group showed that it provides a moderate analgesic benefit for 3 months [3]. Some studies reported that DRG-PRF therapy resulted in significant improvement in NRS and Oswestry disability index (ODI) scores for 4 months in patients with chronic LRP [4]. A study comparing the efficacy of DRG-PRF and TFESI in the treatment of radicular pain associated with LDH demonstrated improvements in visual analog scale (VAS) and ODI scores for 3 months in both groups with no statistically significant difference between them [10]. There are also studies in the literature examining whether the combined use of TFESI and DRG-PRF increases treatment success compared to TFESI alone. In a study comparing the efficacy of combined TFESI and DRG-PRF with TFESI alone, the TFESI and DRG-PRF group showed significantly greater improvement in NRS and ODI scores compared to the TFESI group for 3 months [2]. In another study comparing the efficacy of combined TFESI and DRG-PRF with TFESI alone, combined therapy was reported to result in significantly greater improvement in VAS score compared to TFESI alone for the first 3 months, but this effect disappeared at 4 months [5]. Therefore, the patients in the present study were treated with TFESI and DRG-PRF together in order to increase the effectiveness of TFESI. Significant improvement in NRS scores for 3 months was observed in 66.6% of patients who underwent combined TFESI and DRG-PRF therapy.

Ekedahl et al. reported in their study that younger age (53–60 years) was a significant predictor of a favorable response within 1 year after TFESI [6]. However, the results of another study indicated that patients aged 60–69 years showed higher success after TFESI [11], while other studies suggested that patient age has no effect on TFESI treatment success [12]. In a study following patients for 6 months after DRG-PRF treatment, more successful results were obtained in patients aged ≥55 years [9]. In another study examining the efficacy of DRG-PRF in chronic LRP, age >57 years was evaluated as a negative prognostic factor for treatment success [13]. In the present study, there was no significant association between patient age and the 3-month results of combined TFESI and DRG-PRF therapy. Studies including a wider range of age groups are needed to elucidate the age factor. A study examining the impact of sex on LRP prognosis concluded that women had a 3.3-fold poorer prognosis than men within the first year [14]. In another study of prognostic factors associated with the success of DRG-PRF therapy, 72.3% of the patients were women [9]. In the present study, women comprised 60.4% of the sample and no difference in sex distribution was observed between patients with treatment success and failure at 3 months after combined TFESI and DRG-PRF therapy.

Some authors have argued that shorter duration of radicular pain symptoms has a favorable impact on treatment success with TFESI [6]. In contrast, no correlation between symptom duration and TFESI success was observed in other studies [12]. However, there is no previous study in the literature investigating the relationship between DRG-PRF treatment for radicular pain and the duration of symptoms. In the present study, there was no difference in symptom duration between patients with treatment success and failure. Further studies with patient groups that have different symptom durations are needed.

In a study examining the association between MRI and fluoroscopic image findings and the success of TFESI therapy, the procedure was performed most frequently at L4–5 and second most frequently at L5–S1 [15]. In another study to identify factors predicting the treatment success of TFESI for LRP, the two most common procedure sites were L5–S1 and the S1 foramen, respectively [12]. The authors of both studies concluded that procedure level had no effect on treatment success. In this study, procedures were most commonly performed at L5–S1, followed by L4–5, and more frequently on the left side. Injection level and side were not predictive of treatment success. Larger scale studies are needed to obtain more accurate data.

Neuropathic pain after spinal surgery, called failed back surgery syndrome (FBSS), is a common but difficult-to-treat condition. Although the efficacy of spinal cord stimulation in FBSS has been established in the literature, epidural injections and DRG-PRF have been recommended as a first-line treatment because they are easy to perform and rarely cause complications [16]. Patients with FBSS who received epidural steroid injections were shown to improve for the first month, after which the effect of treatment decreased at 3- and 6-month follow-up [17]. In another study, 2 out of 3 patients with FBSS who underwent DRG-PRF had a fair to good improvement in pain during a 6-month follow-up period, with 1 patient reporting only short-term pain relief [18]. The results of a study assessing the efficacy of DRG-PRF in patients with LDH, spinal stenosis, and FBSS suggested that DRG-PRF was ineffective in FBSS [8]. Although patients in the present study who had undergone at least one spinal surgery were more likely to have a treatment failure, history of spinal surgery was not statistically associated with treatment success. In order to provide clearer information on this subject, prospective randomized controlled studies with more patients and longer follow-ups are needed.

In a study to identify predictors of treatment success with TFESI in LDH-induced radicular pain, the authors reported that a greater reduction in NRS pain scores at 1 h after the procedure was predictive of a favorable response for 3 months after treatment, and they concluded that 1-h NRS pain scores could be a useful marker for identifying patients who will benefit from treatment [12]. In the present study, a greater reduction in NRS scores 10 days after combined TFESI and DRG-PRF therapy was able to predict a favorable treatment response at 3 months. Because day-10 NRS score is not a pretreatment factor, it will not contribute to the prediction of treatment success before the procedure. Nevertheless, predicting treatment success after 10 days seems valuable for the patient and the physician in terms of providing objective information about the success of the procedure.

One of the limitations of this study is that diagnostic selective nerve root block was not performed. In the literature, one study comparing the effectiveness of TFESI alone and combined with DRG-PRF involved preoperative diagnostic TFESI, while no diagnostic test was performed in another study [2,5]. Another limitation of this study is the short-term follow-up and retrospective design. Randomized controlled studies are needed to determine whether this intervention is more effective in patients with certain clinical characteristics.

## 6. Conclusion

This study showed that treatment success with combined TFESI and DRG-PRF was not associated with patient age, sex, symptom duration, procedure site, or history of spinal surgery. However, the more favorable prognosis among patients with a significant decrease in NRS score on day 10 is a finding that may shed light on the follow-up process. The results of this study may serve as a guide for future research.

## Figures and Tables

**Table 1:** Comparison of demographic characteristics, treatment details, and follow-up data between patients with successful and unsuccessful treatment.

	Treatment outcome		p value
	Successful (n = 32)	Unsuccessful (n = 16)	
**Age (years)**	51.72 ± 13.83	51.19 ± 12.63	0.898
**Sex**			
Female	20 (62.50%)	9 (56.30%)	0.676
Male	12 (37.50%)	7 (43.80)	
**Symptom duration (months)**	6 (8.50)	8.50 (18.75)	0.109
**Injection level**			
L4–5	15 (46.90%)	3 (18.80%)	0.102
L5–S1	14 (43.80%)	9 (56.30%)	
L4–5 and L5–S1	1 (3.10%)	3 (18.80%)	
L5–S1 and S1 foramen	2 (6.30%)	1 (6.30%)	
**Injection side**			
Right	8 (25%)	5 (31.30%)	0.917
Left	18 (56.30%)	9 (56.30%)	
Bilateral	6 (18.80%)	2 (12.50%)	
**History of spinal surgery** *			
None	27 (87.10%)	4 (12.90%)	**<0.001**
Once	4 (33.30%)	8 (66.70%)	
Twice or more	1 (20%)	4 (80%)	
**NRS**			
Pretreatment	6 (0)	6 (1.75)	0.207
Posttreatment day 10	1.50 (1)	4 (3)	-
Posttreatment 1 month	2 (1.50)	5.50 (2)	-
Posttreatment 3 months	2 (0.75)	5.50 (3)	-
Day 10 – Pretreatment	−5 (1)	−2 (2)	**<0.001**
1 month – Pretreatment	−4 (1)	−1.50 (1.75)	**<0.001**
3 months – Pretreatment	−4 (1)	−1 (2)	**<0.001**

Data are expressed as mean ± standard deviation, median (interquartile range), or n (%).

* Percentage based on spinal surgery variable.

**Table 2:** Analysis of factors associated with treatment success.

	Wald	OR (95% CI)	p value
**Symptom duration**	2.64	0.83 (0.67–1.04)	0.105
**Previous spinal surgery (Ref cat: ≥2)**			
0	0.46	0.28 (0.01–11.16)	0.499
1	2.83	0.01 (0–2.64)	0.093
**NRS**			
Pretreatment	5.22	0.28 (0.10–0.84)	**0.022**
Posttreatment day 10 – pretreatment	7.04	0.05 (0.01–0.45)	**0.008**

Model χ^2^=51.08, **p < 0.001**.

Hosmer–Lemeshow test: p = 0.987.

OR: Odds ratio, CI: Confidence interval, Ref cat: Reference category.

**Table 3:** Comparison of age, symptom duration, and NRS scores between patients with and without spinal surgery.

	History of spinal surgery		P value
	Yes (n = 21)	No (n = 27)	
**Age (years)**	51.67 ± 13.04	51.44 ± 13.76	0.955
**Symptom duration (months)**	8 (18.50)	6 (7)	0.077
**NRS**			
Pretreatment	6 (1.50)	6 (0)	0.292
Posttreatment day 10	4 (3.50)	2 (1)	-
Posttreatment month 1	4 (2.50)	2 (1)	-
Posttreatment month 3	5 (2.50)	2 (0)	-
Posttreatment day 10 – Pretreatment	−3 (3)	−5 (1)	**<0.001**
Posttreatment month 1 – Pretreatment	−2 (2)	−4 (1)	**<0.001**
Posttreatment month 3 – Pretreatment	−2 (2.50)	−4 (1)	**<0.001**

Data are expressed as mean ± standard deviation, median (interquartile range), or n (%).
